# Citrate anticoagulation for CRRT: don’t always trust the postfilter iCa results!

**DOI:** 10.1186/s13054-015-1148-6

**Published:** 2015-12-04

**Authors:** Heleen M. Oudemans-van Straaten, Marlies Ostermann

**Affiliations:** Department of Adult Intensive Care, VU University Medical Centre, De Boelelaan 1118, 1081 HZ Amsterdam, The Netherlands; Department of Critical Care and Nephrology, King’s College London, Guy’s and St Thomas’ Hospital, London, SE1 7EH UK

## Abstract

Citrate has been recommended as the first-line anticoagulant for continuous renal replacement therapy (CRRT) in critically ill patients. Compared with heparin, citrate anticoagulation is safer and more efficacious. Citrate inhibits the coagulation cascade by lowering the ionized calcium (iCa) concentration in the filter. Monitoring of systemic iCa concentrations is inherent to the protocol, and monitoring of postfilter iCa is recommended to adjust citrate flow and optimize anticoagulation. While systemic iCa targets are in the physiological range, postfilter iCa concentrations are targeted between 0.20 and 0.35 mmol/l. In a previous issue of *Critical Care*, Schwarzer et al. compared systemic and postfilter iCa measurements of patients receiving citrate-based CRRT between six devices. They highlight the unreliability of iCa concentrations in the postfilter range, because the instruments cannot be validated in the low iCa range. The maximum mean difference between two instruments was as high as 0.33 mmol/l (range 0.21–0.50 mmol/l). The authors call for dialysis companies to revise their protocols. However, the first implication of their study is that the accuracy of blood gas analyzers to measure iCa in the low range needs to improve; and, secondly, clinicians using citrate anticoagulation need to be aware that the postfilter iCa result may be falsely high or low. This is particularly relevant when frequent premature filter clotting is observed despite postfilter iCa results in the seemingly target range. In these situations, citrate flow can be safely increased up to 4 mmol/l blood flow under monitoring of signs of citrate accumulation.

## Introduction

Citrate has been recommended as the first-line anticoagulant for continuous renal replacement therapy (CRRT) in critically ill patients. Citrate inhibits the coagulation cascade by lowering the ionized calcium (iCa) concentration through the chelation of calcium in the filter. A proportion of the calcium citrate complexes is removed via the filter and the remainder enters the systemic circulation where citrate is rapidly metabolized. The calcium lost in the effluent has to be replaced. Monitoring of systemic iCa concentration to guide this replacement is crucial to the safe application of citrate. In some protocols, monitoring of postfilter iCa is also recommended to adjust citrate flow and optimize anticoagulation.

In a recent issue of *Critical Care*, Schwarzer et al. [1] highlight a potential problem related to the measurement of postfilter iCa. They measured iCa levels in systemic and postfilter blood from patients undergoing citrate-based CRRT using six different blood gas analyzers and found concordance of the systemic iCa results, but marked discrepancies between postfilter iCa concentrations. Clinical protocols recommend targeting postfilter iCa concentrations between 0.20 and 0.35 mmol/l, because the anticoagulant effect begins when iCa falls below 0.50 mmol/l and is complete at 0.25 mmol/l [2]. In the Schwarzer et al. study, the maximum mean difference between two instruments was 0.33 mmol/l (median 0.29 mmol/l, range 0.21–0.50 mmol/l) for postfilter iCa values despite internal quality controls within the 14 % variation of combined imprecision and bias according to national regulation. It appears that modern blood gas analyzers are not designed or validated to measure iCa concentrations outside the physiologic range. They are nevertheless used for this purpose in clinical practice. Importantly, reference methods for measuring low iCa, as targeted during citrate anticoagulation in the extracorporeal circuit, are lacking.

Because the reported potential margin of error in the postfilter iCa results is unacceptably high, the authors suggest that postfilter iCa monitoring may be questionable. They also warn about the potential risk of citrate accumulation as a result of erroneous and misleading postfilter iCa results and call for a revision of existing citrate protocols. Although the authors should be congratulated for highlighting a potentially serious problem, we do not fully agree with all of their conclusions. The potential danger of citrate toxicity as a result of misleading iCa results is exaggerated.

Citrate has been recommended as the first-line anticoagulant for CRRT in critically ill patients [3]. Compared with heparin, citrate anticoagulation is safer and confers less bleeding, longer circuit life, and less circuit downtime [4–7]. The protocols used in these studies are currently used worldwide, and have shown superiority over heparin anticoagulation [8], despite using the potentially inaccurate devices for iCa measurement. Nevertheless, if postfilter iCa monitoring is part of the protocol, centers may presently base their decision on either falsely low, falsely high, or accurate postfilter iCa concentrations. The question arises as to whether this poses unnecessary risks for patients. If the results are falsely low, the citrate dose may be too low for optimal anticoagulation and this may induce early filter clotting or reduce the sodium and buffer supply to the patient. If the iCa results are falsely high, the adjusted citrate dose may be higher than needed for optimal anticoagulation in which case more citrate enters the systemic circulation. Is this a problem? Yes, possibly, but only in a small proportion of patients. It may confer a trend to hypernatremia and alkalosis if citrate metabolism is sufficient, or may increase the risk of accumulation if metabolism is limited. However, the overall incidence of accumulation is low (i.e., around 3 % [9]), with patients with acute liver failure or decompensated liver cirrhosis being at increased risk. Fortunately, citrate accumulation can easily be monitored by measuring the total/iCa ratio in the systemic circulation [10].

The authors call for dialysis companies to revise their protocols. However, in our opinion, the manufacturers of blood gas analyzers should be prompted to improve the accuracy of their devices in the lower than physiological iCa levels. This is particularly important in light of the rising worldwide trend to use citrate-based CRRT. It should also be noted that the authors only tested the accuracy of blood gas analyzers, but not their precision. Precision relates to reproducibility and describes the degree to which repeated measurements show the same result. It remains unclear whether the individual blood gas analyzers were consistently under-reading or over-reading.

The two main implications of the study by Schwarzer et al. are as follows: firstly, the accuracy of blood gas analyzers to measure iCa in the low range needs to improve; and, secondly, clinicians using citrate anticoagulation need to be aware that the postfilter iCa result may not be accurate. This is particularly relevant when frequent premature filter clotting is observed despite postfilter iCa results in the seemingly target range. It should also be recognized that monitoring iCa concentrations is not necessarily required because some protocols use a fixed relation between citrate and blood flow (summarized in [8]). Targeting citrate concentration up to 4 mmol/l blood flow has proven to be safe.

## Conclusions

The study by Schwarzer et al. shows that postfilter iCa monitoring of regional anticoagulation with citrate may be unreliable, because the measurement cannot be validated in the low iCa range. As a result, anticoagulation of the extracorporeal circuit may be suboptimal. Until reference methods for low iCa in whole blood containing citrate are available, clinicians have good reasons to mistrust postfilter iCa results, especially in the case of otherwise unexplained early filter clotting. In these situations, citrate flow can be safely increased up to 4 mmol/l blood flow, or higher under close monitoring of signs of citrate accumulation and acid–base balance.

## Abbreviations

CRRT: Continuous renal replacement therapy
iCa: Ionized calcium

## References

[1] Schwarzer P, Kuhn S-O, Stracke S, Grünling M, Knigge S, Selleng S, Helm M (2015). Discrepant post filter ionized calcium concentrations by common blood gas analyzers in CRRT using citrate anticoagulation. Crit Care.

[2] Calatzis A, Toepfer M, Schramm W, Spannagl M, Schiffl H (2001). Citrate anticoagulation for extracorporeal circuits: effects on whole blood coagulation activation and clot formation. Nephron.

[3] Kidney Disease: Improving Global Outcomes (KDIGO) Acute Kidney Injury Work Group (2012). KDIGO Clinical Practice Guideline for Acute Kidney Injury. Kidney Int Suppl.

[4] Zhang Z, Hongying N (2012). Efficacy and safety of regional citrate anticoagulation in critically ill patients undergoing continuous renal replacement therapy. Intensive Care Med.

[5] Schilder L, Nurmohamed SA, Bosch FH, Purmer IM, den Boer SS, Kleppe CG (2014). Citrate anticoagulation versus systemic heparinisation in continuous venovenous hemofiltration in critically ill patients with acute kidney injury: a multi-center randomized clinical trial. Crit Care.

[6] Stucker F, Ponte B, Tataw J, Martin PY, Wozniak H, Pugin J, Saudan P (2015). Efficacy and safety of citrate-based anticoagulation compared to heparin in patients with acute kidney injury requiring continuous renal replacement therapy: a randomized controlled trial. Crit Care.

[7] Oudemans-van Straaten HM, Kellum JA, Bellomo R (2011). Clinical review: anticoagulation for continuous renal replacement therapy—heparin or citrate?. Crit Care.

[8] Oudemans-van Straaten HM, Ostermann M (2012). Bench-to-bedside review: Citrate for continuous renal replacement therapy, from science to practice. Crit Care.

[9] Khadzhynov D, Schelter C, Lieker I, Mika A, Staeck O, Neumayer HH (2014). Incidence and outcome of metabolic disarrangements consistent with citrate accumulation in critically ill patients undergoing continuous venovenous hemodialysis with regional citrate anticoagulation. J Crit Care.

[10] Schultheiss C, Saugel B, Phillip V, Thies P, Noe S, Mayr U (2012). Continuous venovenous hemodialysis with regional citrate anticoagulation in patients with liver failure: a prospective observational study. Crit Care.

